# Explainable MRI radiomics identifies a PTEN-associated imaging phenotype in adult gliomas

**DOI:** 10.3389/fonc.2026.1900609

**Published:** 2026-08-19

**Authors:** Rafail C. Christodoulou, Georgios Vamvouras, Mateus A. Esmeraldo, Platon S. Papageorgiou, Evros Vassiliou, Elena E. Solomou, Sokratis G. Papageorgiou, Michalis F. Georgiou

**Affiliations:** 1 Division of Neuroimaging and Neurointervention, Department of Radiology, Stanford University, Stanford, CA, United States; 2 Department of Electrical and Computer Engineering, National Technical University of Athens NTUA, Athens, Greece; 3 Department of Radiology, Stanford University, Stanford, CA, United States; 4 Department of Medicine, National and Kapodistrian University of Athens, Athens, Greece; 5 Department of Biological Sciences, Kean University, Union, NJ, United States; 6 Internal Medicine-Hematology, University of Patras Medical School, Rion, Greece; 7 1st Department of Neurology, Medical School, National and Kapodistrian University of Athens, Eginition Hospital, Athens, Greece; 8 Department of Radiology, University of Miami, Miami, FL, United States

**Keywords:** explainable AI, gliomas, machine learning, molecular biomarker, PTEN (phosphatase and tensin homolog deleted on chromosome 10), radiomics

## Abstract

**Introduction:**

PTEN mutation is a recurrent molecular alteration in adult gliomas, associated with PI3K/AKT/mTOR pathway activation, aggressive tumor behavior, and resistance to therapy. PTEN status is currently determined through tissue biopsy, which is susceptible to sampling bias and intratumoral heterogeneity. We investigated whether conventional multiparametric MRI radiomics can identify a noninvasive, interpretable PTEN-associated imaging phenotype in adult gliomas.

**Methods:**

We retrospectively analyzed 195 adult glioma patients with known PTEN mutation status (184 PTEN-negative, 11 PTEN-positive) from the TCIA MU-Glioma-Post database. A total of 3,669 radiomic features were extracted from enhancing tumor core segmentations on T1CE, T2, and FLAIR MRI. A leakage-conscious nested cross-validation pipeline (5 outer/3 inner folds, 30 Optuna trials) was used for redundancy-aware feature selection, imbalance-adjusted model tuning, and performance evaluation across four classifier architectures. Feature-level explainability was assessed using linear SHAP-style additive contributions. *Post-hoc* Firth-penalized logistic regression was performed to assess whether the radiomic association was explained by age, IDH status, MRI timing, or treatment exposure.

**Results:**

The primary elastic-net logistic regression classifier achieved an outer out-of-fold ROC-AUC of 0.874 (95% CI 0.808–0.936), sensitivity of 90.9%, specificity of 71.7%, balanced accuracy of 81.3%, and Brier score of 0.052. PR-AUC was 0.211 (95% CI, 0.112–0.400), reflecting a low PTEN-positive prevalence (5.6%) and indicating limited positive predictive value despite strong discrimination. The most influential features were wavelet- and LoG-filtered GLCM and NGTDM texture descriptors from T1CE, T2, and FLAIR, suggesting a multisequence phenotype characterized by heterogeneous enhancement architecture and disrupted local texture organization. The radiomic score remained independently associated with PTEN positivity after adjustment for age, MRI timing, and documented treatment exposure across multiple Firth-penalized models (OR per SD range: 1.57–1.62, all p ≤ 0.005).

**Discussion:**

Conventional multiparametric MRI radiomics identifies a biologically plausible, explainable PTEN-associated imaging phenotype in adult gliomas that is robust to adjustment for clinical and treatment-related confounders. However, given the small PTEN-positive cohort, severe class imbalance, modest PR-AUC, and absence of external validation, the findings are exploratory and hypothesis-generating. Prospective multi-institutional validation in larger, molecularly balanced cohorts is required before clinical translation.

## Graphical Abstract

**Figure:**
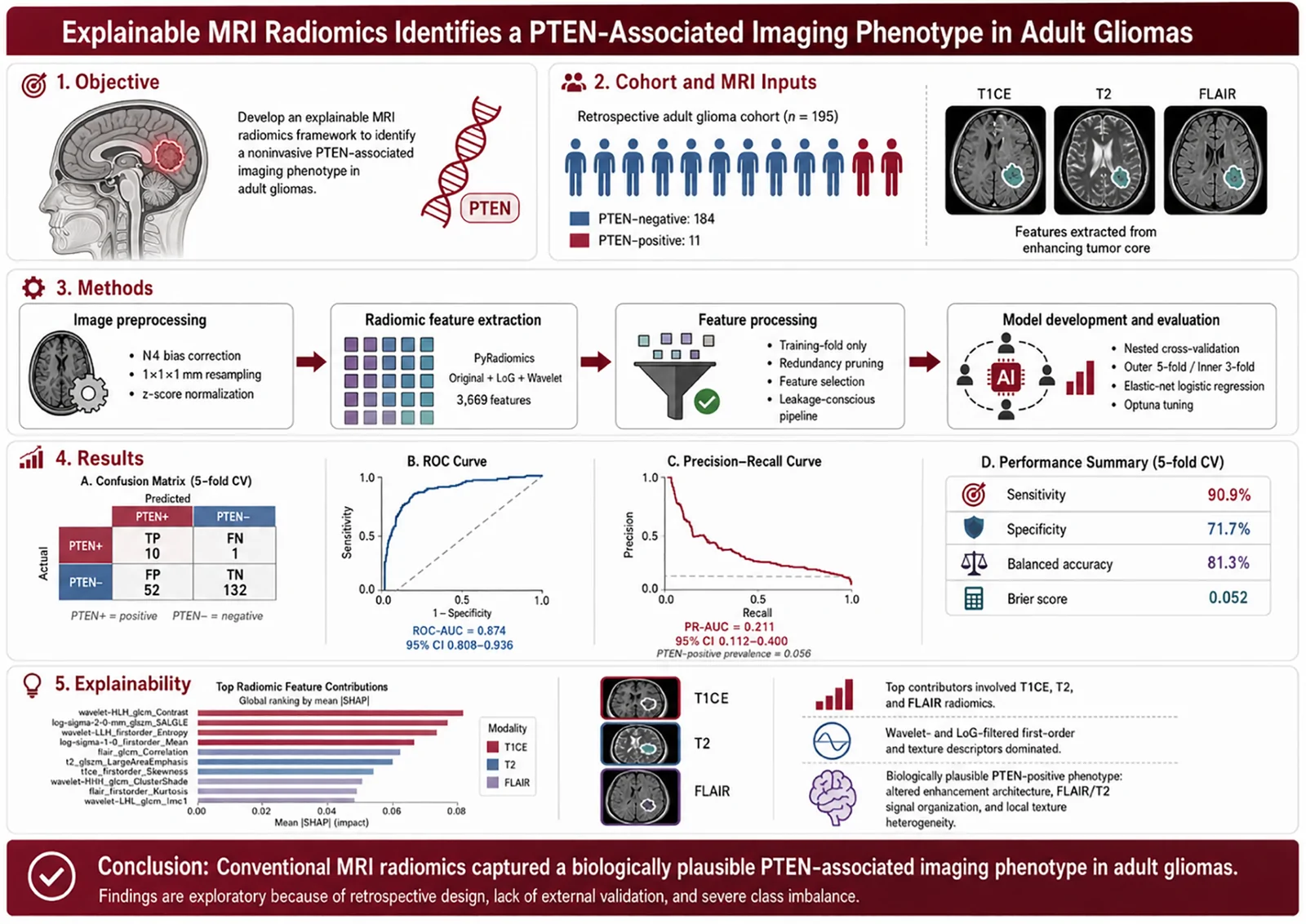

## Introduction

Gliomas are the most common primary malignant tumors of the central nervous system and arise from glial cells (1). The 2021 WHO classification of the central nervous system introduced an integrated histomolecular classification in which gliomas are classified using both histopathologic and molecular features. IDH mutation status and 1p/19q codeletion are central diagnostic markers that distinguish IDH-mutant astrocytoma, IDH-mutant and 1p/19q codeleted oligodendroglioma, and IDH-wildtype glioblastoma (2). Additional molecular alterations, including MGMT promoter methylation, TERT promoter mutation, EGFR amplification, ATRX alteration, and CDKN2A/B deletion, provide important diagnostic, prognostic, and treatment-relevant information (3). Despite progress in molecular classification and treatments, high-grade gliomas, especially glioblastoma, continue to be linked with low survival rates, significant spatial heterogeneity, and limited long-lasting responses to therapy (4). United States population data revealed that the survival rate for patients with malignant brain and other central nervous system tumors has remained low, approximately at 35% (5).

Mutations in the PTEN gene play a critical role in glioma growth. This importance is directly related to PTEN’s function as a protein and lipid phosphatase, which impacts the PI3K/AKT/mTOR signaling pathway and specifically acts as a tumor suppressor gene in this pathway. PTEN is involved in essential cellular processes such as cell cycle regulation, migration, growth, and apoptosis (6). Interestingly, AKT inhibitors have shown promising results in preclinical glioblastoma treatment models (7). In clinical practice, detecting PTEN mutations is important because they are associated with poorer overall survival and treatment resistance, particularly chemotherapy and immunotherapy (8–10). Currently, PTEN status is determined solely through tissue-based molecular testing, which is limited by intratumoral spatial heterogeneity, meaning PTEN alterations may be present only in certain tumor areas and could be missed by a single biopsy. Additionally, the procedure carries risks and is often not feasible to repeat at the time of recurrence, a critical time for treatment decisions. Recognizing PTEN mutation status as an important stratification factor in clinical trials targeting the PI3K/AKT/mTOR pathway emphasizes the need for non-invasive, repeatable molecular assessments using routine MRI (11, 12). This approach is especially valuable in post-treatment surveillance, where re-biopsy is rarely done but adjustments in therapy are often essential.

Radiomics offers a noninvasive approach to quantifying tumor phenotype by extracting high-dimensional intensity, shape, and texture features from routine imaging (13). In glioma imaging, radiomics have been used to predict molecular alterations, tumor grade, treatment response, and patient survival (14, 15). Prior research studies have also explored MRI-based prediction of PTEN mutation status, including deep learning and radiomics approaches, suggesting that PTEN mutation may produce an imaging phenotype detectable on conventional MRI (11). Nevertheless, the existing PTEN radiogenomic literature remains limited, and it is unclear whether conventional multiparametric MRI radiomics can identify a reproducible and interpretable PTEN-associated phenotype (12). Previous PTEN radiogenomic research mainly used preoperative imaging, basic train/test splits without nested validation, and either single or dual-sequence inputs. These studies did not address issues such as feature redundancy, class imbalance, or data leakage in high-dimensional data (11, 12, 16). No previous study has examined PTEN-related radiomic features in post-treatment surveillance imaging, provided feature-level explanations linking radiomic data to biological mechanisms, or formally tested whether the radiomic associations remain consistent when accounting for clinical confounders such as age, IDH status collinearity, MRI timing, and treatment exposure.

Our primary objective was to determine whether conventional multiparametric MRI radiomics can identify a noninvasive, interpretable imaging phenotype associated with PTEN mutation status in adult gliomas and to assess whether this association is robust to clinical and treatment-related confounding. A secondary objective was to characterize the contributing radiomic features through explainability analysis, linking imaging-derived signals to biologically plausible mechanisms underlying PTEN-associated tumor heterogeneity. To evaluate these objectives, we hypothesized that PTEN-mutant gliomas exhibit a distinct multisequence radiomic profile in the enhancing tumor core, characterized by differences in enhancement heterogeneity, edema-related signals, and texture organization.

## Materials and methods

### Study design and cohort

This retrospective radiogenomic study examined whether multiparametric MRI radiomics can detect an imaging phenotype associated with PTEN mutation status in an adult glioma cohort (WHO Grades 1-4) from the TCIA MU Glioma Post database (17). The primary radiomics analysis focused on patients with an available segmentation mask of the enhancing tumor core, chosen because it is believed to be the most biologically active part of the tumor and has shown the strongest radiomic signals in similar glioma studies (18). The final cohort consisted of 195 patients, including 184 PTEN-negative and 11 PTEN-positive cases. PTEN mutation status was treated as a binary endpoint: PTEN-negative was defined as PTEN mutation = 0 (no mutation), and PTEN-positive as PTEN mutation = 1 (mutation present). Throughout this manuscript, “PTEN-positive” refers specifically to tumors with documented PTEN mutation in the dataset, and “PTEN-negative” refers to tumors without documented PTEN mutation. Broader terms such as PTEN alteration or PTEN pathway dysregulation are used only when discussing general biological mechanisms, not the binary endpoint analyzed in this study. Baseline clinical and molecular patient characteristics are summarized in **Table 1**. Furthermore, additional dataset characteristics, including MRI timing, treatment exposure, and others, are provided in **Supplementary Table 1**.

**Table 1:** Cohort characteristics stratified by PTEN status.

Characteristic	Category	PTEN-negative, n =184	PTEN-positive, n = 11	P value
Age at diagnosis	Median [IQR], years	59 [48–69]	66 [63–73]	0.028
	Mean ± SD, years	57.3 ± 16.4	68.5 ± 8.0	
Sex at birth	Female	74/184 (40.2%)	3/11 (27.3%)	0.532
	Male	110/184 (59.8%)	8/11 (72.7%)	
IDH status	Mutant	26/184 (14.1%)	0/11 (0.0%)	0.402
	Unknown/indeterminate	14/184 (7.6%)	0/11 (0.0%)	
	Wildtype	144/184 (78.3%)	11/11 (100.0%)	
MGMT promoter status	Methylated	59/184 (32.1%)	6/11 (54.5%)	0.377
	Unmethylated	87/184 (47.3%)	5/11 (45.5%)	
	Indeterminate	11/184 (6.0%)	0/11 (0.0%)	
	Unknown/not assessed	27/184 (14.7%)	0/11 (0.0%)	

Values are presented as median [interquartile range], mean ± standard deviation, or n/N (%), as appropriate. P values compare PTEN-positive and PTEN-negative groups. Continuous variables were compared using the two-sided Mann-Whitney U test. Categorical variables were compared using Fisher’s exact tests.

### Image preprocessing and radiomic feature extraction

Radiomic preprocessing and extraction were conducted using PyRadiomics, with N4 bias-field correction enabled. MRI images were resampled to 1 × 1 × 1 mm isotropic resolution using B-spline interpolation for images and nearest-neighbor interpolation for segmentation masks. Images were then z-score normalized within the tumor mask. Radiomic features were extracted with a bin width of 25, image normalization turned on, normalization scale set to 100, and mask correction enabled. Features were derived from both original images and filtered images created with Laplacian-of-Gaussian and wavelet filters, using LoG sigma values of 0.5, 1.0, 2.0, and 3.0 mm. The features included shape, first-order intensity, gray-level co-occurrence matrix, gray-level run-length matrix, gray-level size-zone matrix, gray-level dependence matrix, and neighboring gray-tone difference matrix features. For the primary analysis, 3,669 features were extracted from T1CE, T2, and FLAIR MRI.

### Feature processing and redundancy reduction

All feature preprocessing and selection steps were strictly confined to the training folds to prevent information leakage. The radiomics pipeline involved missingness filtering, near-zero-variance filtering, Spearman correlation-based redundancy pruning, Mann–Whitney U-based filtering, L1-regularized and permutation-based feature-importance ranking, final top-k feature selection, and feature scaling. Spearman correlation pruning aimed to reduce collinearity among radiomic features by removing the lower-ranked feature from highly correlated pairs and retaining the higher-ranked one. This redundancy-aware approach was designed to minimize the impact of highly correlated radiomic descriptors while keeping features more strongly linked to PTEN mutation status. The effectiveness of this filtering was evaluated using feature-correlation heatmaps and upper-tail redundancy metrics.

### Model development

Model development and evaluation were conducted through patient-level nested internal validation. The outer loop used five stratified folds for performance estimation, while the inner loop involved three stratified folds for hyperparameter tuning. Hyperparameters were optimized with Optuna across 30 trials within the inner cross-validation. The primary classifier was an elastic-net-regularized logistic regression model based on T1CE, T2, and FLAIR radiomic features. Comparison models included L2-regularized logistic regression, an Extra Trees classifier, and an ensemble combining elastic-net and Extra Trees. To address class imbalance, only the training folds were downsampled in the majority class; outer validation folds were not resampled. The negative-to-positive sampling ratio was fine-tuned within cross-validation, testing ratios of 1:1, 2:1, 3:1, and 5:1. The final model did not incorporate class weighting.

### Reproducibility and hyperparameter reporting

To enhance reproducibility and align with reporting guidelines, we provided the complete nested cross-validation workflow for feature reduction and tuning in **Supplementary Table 2** (19). In summary, all steps—such as preprocessing, feature filtering, redundancy pruning, feature selection, class imbalance mitigation, scaling, hyperparameter tuning, calibration, and threshold setting—were conducted only within the training folds. The parameters for Spearman correlation threshold, Mann–Whitney feature retention count, final top-k features, negative-to-positive downsampling ratio, and elastic-net hyperparameters were selected independently within each outer training fold using inner cross-validation. No outer-fold test data was used during preprocessing, feature selection, resampling, calibration, or threshold determination. Lastly, the code for the model development pipeline is available at https://github.com/Rafail99/PTEN

### Model evaluation

The primary performance endpoint was the outer out-of-fold ROC-AUC for PTEN-positive versus PTEN-negative classification. Secondary metrics included precision, recall, AUC, Brier score, sensitivity, specificity, balanced accuracy, and confusion-matrix counts. Given the very small PTEN-positive class, precision–recall AUC and confusion-matrix results were interpreted alongside ROC-AUC to account for the marked class imbalance.

### Explainability analysis

Explainability analysis focused on the most informative radiomic features contributing to PTEN-positive classification in the primary elastic-net model. Linear SHAP-style additive contributions were estimated as the standardized radiomic feature value multiplied by the corresponding elastic-net coefficient, representing each feature’s contribution to PTEN-positive log-odds. Features were summarized by MRI modality, image filter, feature class, and feature family.

### Software

Analyses were performed using Python 3.9.6. Radiomic feature extraction was performed using PyRadiomics 3.0.1 and SimpleITK 2.5.4. Machine-learning pipelines were implemented using scikit-learn 1.6.1, and hyperparameter optimization was performed using Optuna 4.8.0.

## Results

### Model configuration and feature reduction

**Figure 1:**
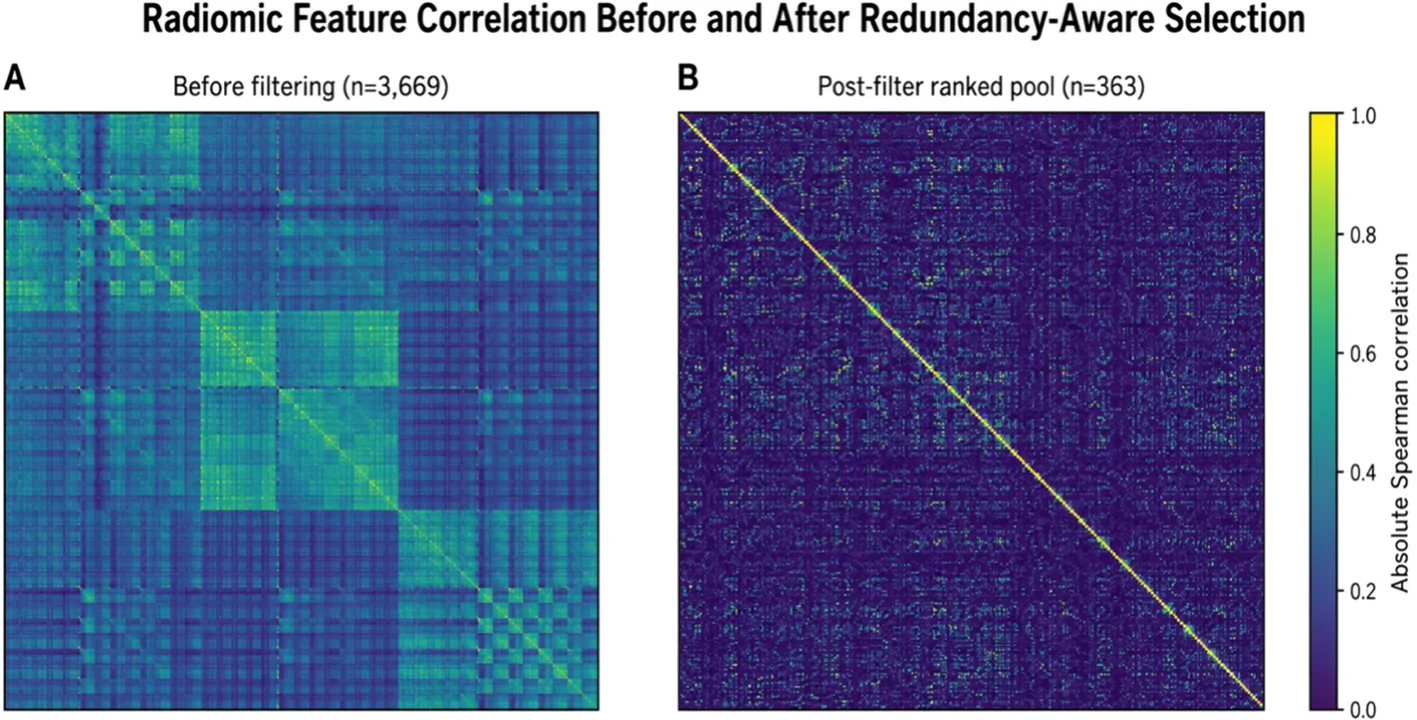
Radiomic feature correlation before and after redundancy-aware feature selection. Absolute Spearman correlation matrices show the radiomic feature space before and after filtering. **(A)** Before filtering, the initial feature pool contained 3,669 features and exhibited a substantial correlation structure, with distinct blocks of highly correlated radiomic descriptors. **(B)** After redundancy-aware filtering, the ranked feature pool was reduced to 363 features, with markedly lower collinearity among the retained features.

**Figure 2:**
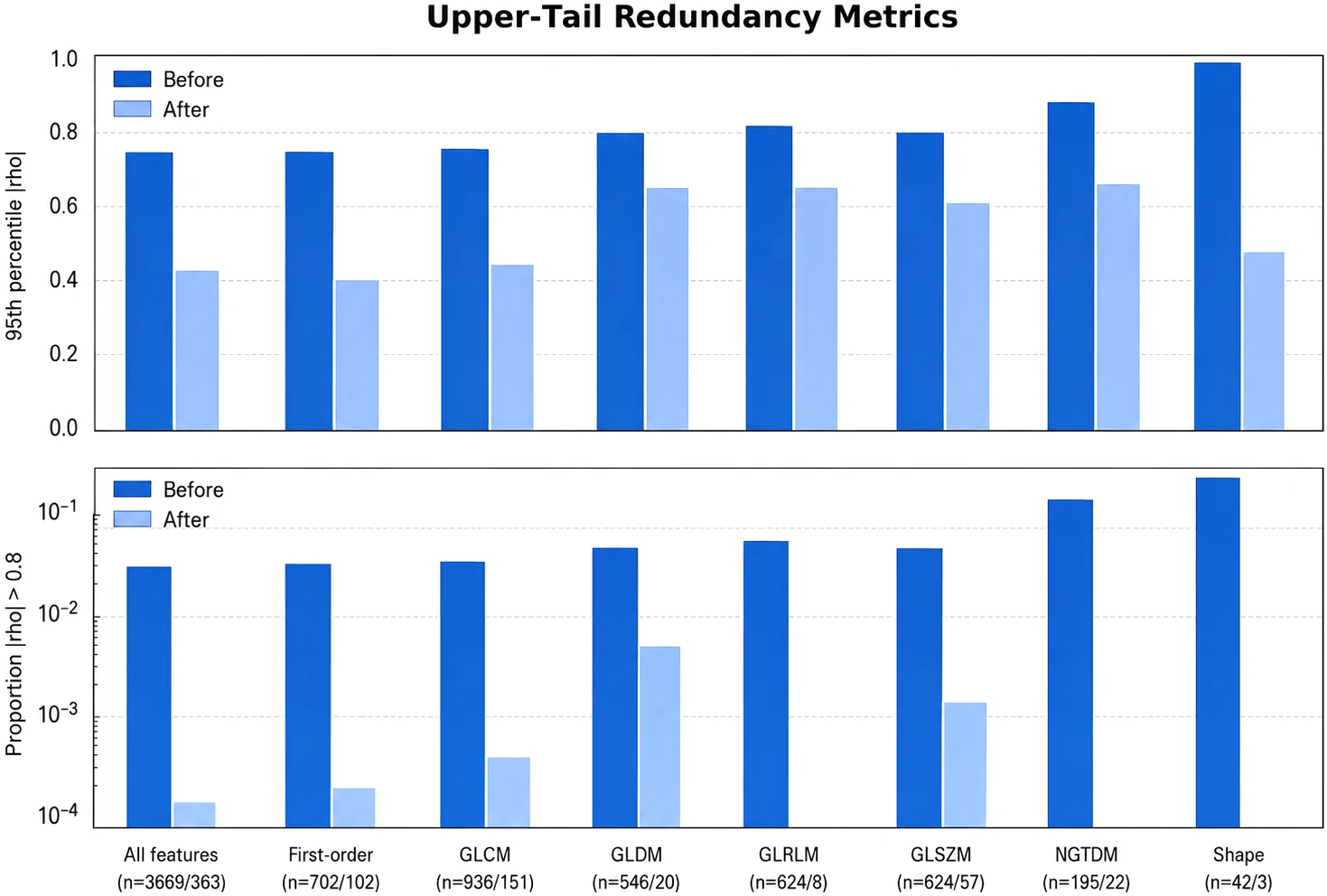
Upper-tail redundancy metrics across radiomic feature families before and after feature filtering. Redundancy was summarized using the 95th percentile of absolute Spearman correlation coefficients and the proportion of feature pairs with absolute Spearman correlation greater than 0.8. Metrics are shown before and after filtering across all features and within major feature families. Filtering substantially reduced high-correlation feature pairs across first-order, GLCM, GLDM, GLRLM, GLSZM, NGTDM, and shape features, confirming that the feature-selection pipeline reduced collinearity while preserving a diverse set of candidate radiomic descriptors.

The initial patient-level radiomic feature set included 3,669 features after aggregating data across available timepoints. Redundancy-aware filtering trimmed this pool to 363 features before the final selection tailored to each model. Correlation analysis initially revealed significant feature redundancy, with visible clusters of highly correlated descriptors among the original 3,669 features. Post-filtering, the ranked feature pool exhibited substantially lower collinearity, supporting the use of a smaller, less redundant feature set for model building (**Figure 1**). Redundancy metrics from the upper tail confirmed this reduction: the 95th percentile of absolute Spearman correlation decreased after filtering, and the share of feature pairs with correlations above 0.8 declined significantly. These reductions were consistent across major feature families, including first-order, GLCM, GLDM, GLRLM, GLSZM, NGTDM, and shape features (**Figure 2**).

### Nested cross-validated model performance

Among the evaluated models, the T1CE/T2/FLAIR elastic-net logistic regression classifier demonstrated the strongest overall discrimination and stability (**Table 2**). The model achieved an outer out-of-fold ROC-AUC of 0.874, with a 95% confidence interval of 0.808 to 0.936. The corresponding inner-CV ROC-AUC used during model tuning was 0.889 ± 0.026.

**Table 2:** Performance of PTEN classification models.

Model	Inner-CV ROC-AUC, mean ± SD	Outer OOF ROC-AUC (95% CI)	OOF PR-AUC (95% CI)	Brier score	Sensitivity (95% CI)	Specificity (95% CI)	Balanced accuracy (95% CI)	PPV (95% CI)	NPV (95% CI)	TP	FP	TN	FN
Elastic net logistic regression	0.889 ± 0.026	0.874 (0.808-0.936)	0.211 (0.112-0.400)	0.052	0.909 (0.700-1.000)	0.717 (0.651-0.783)	0.813 (0.702-0.884)	0.161 (0.076-0.259)	0.992 (0.976-1.000)	10	52	132	1
L2-regularized logistic regression	0.889 ± 0.018	0.800 (0.621-0.944)	0.260 (0.115-0.535)	0.052	1.000 (1.000-1.000)	0.728 (0.665-0.791)	0.864 (0.832-0.896)	0.180 (0.089-0.281)	1.000 (1.000-1.000)	11	50	134	0
Elastic net/Extra Trees ensemble	0.912 ± 0.028	0.715 (0.488-0.910)	0.245 (0.082-0.549)	0.053	0.727 (0.429-1.000)	0.821 (0.762-0.872)	0.774 (0.621-0.906)	0.195 (0.080-0.318)	0.981 (0.955-1.000)	8	33	151	3
Extra Trees classifier	0.903 ± 0.032	0.649 (0.441-0.837)	0.144 (0.059-0.344)	0.053	0.909 (0.700-1.000)	0.696 (0.628-0.762)	0.802 (0.690-0.872)	0.152 (0.070-0.242)	0.992 (0.975-1.000)	10	56	128	1

The elastic-net model achieved an out-of-fold PR-AUC of 0.211, with a 95% confidence interval of 0.112 to 0.400, and a Brier score of 0.052. At the selected operating threshold, sensitivity for PTEN-positive cases was 90.9%, specificity for PTEN-negative cases was 71.7%, and balanced accuracy was 81.3%. The confusion matrix included 10 true positives, 52 false positives, 132 true negatives, and 1 false negative (**Table 2**).

**Figure 3:**
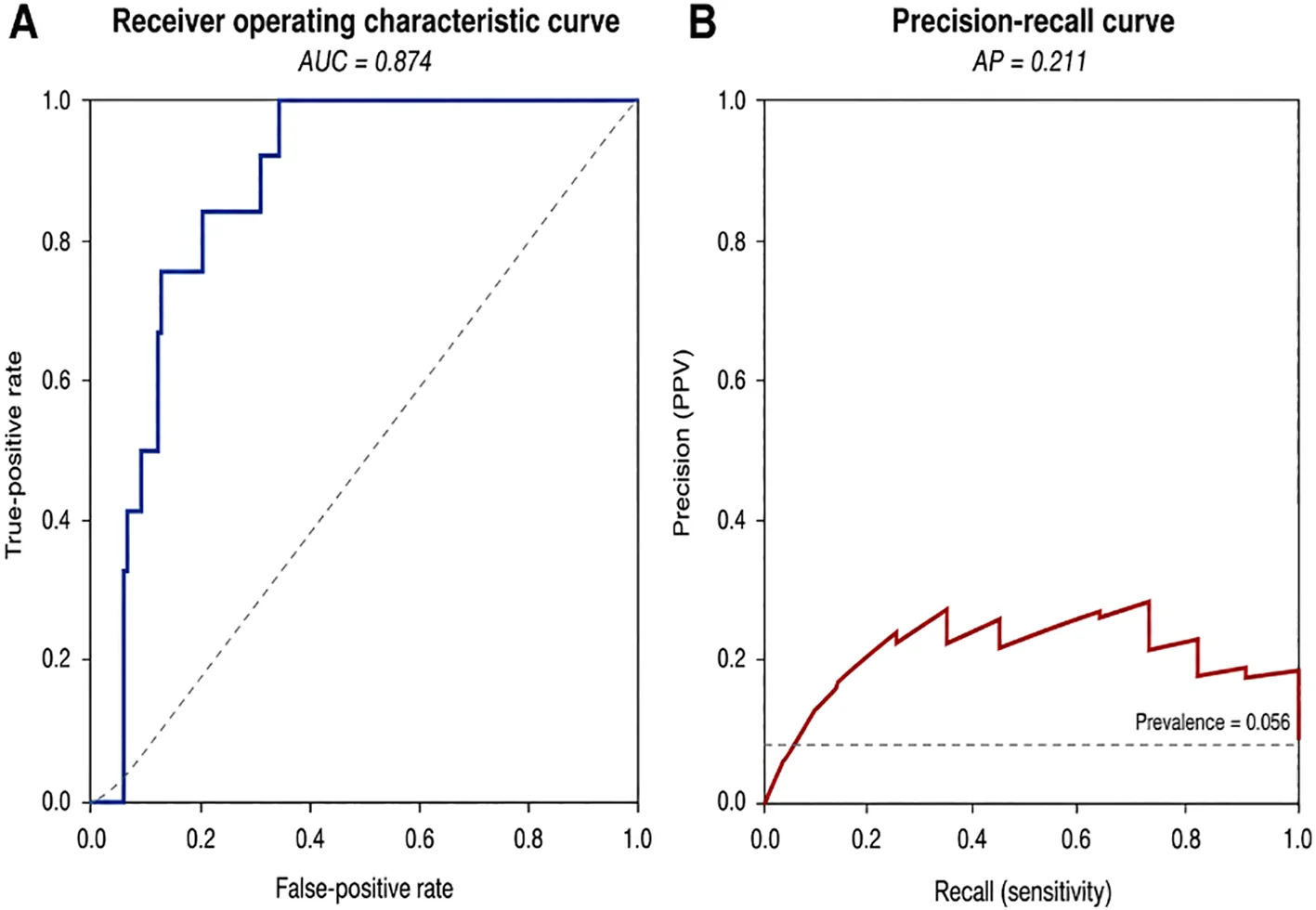
Receiver operating characteristic and precision–recall performance of the elastic-net logistic regression model for PTEN mutation classification. **(A)** Receiver operating characteristic curve based on pooled outer out-of-fold predictions from the nested cross-validation framework. The primary elastic-net model achieved an ROC-AUC of 0.874 for differentiating PTEN-positive from PTEN-negative glioma cases. **(B)** Precision–recall curve for the same pooled out-of-fold predictions. The model achieved an average precision of 0.211, with a PTEN-positive prevalence of 0.056.

The ROC curve showed strong overall discrimination, whereas the precision–recall curve showed more modest performance, with an average precision of 0.211 given a PTEN-positive prevalence of 0.056 (**Figure 3**). This divergence between ROC-AUC and PR-AUC reflects the marked class imbalance in the cohort and supports cautious interpretation of the model’s clinical readiness.

### Classifier architecture comparison

The L2-regularized logistic regression model achieved the highest sensitivity and balanced accuracy, successfully identifying all PTEN-positive cases. It recorded a sensitivity of 100.0%, specificity of 72.8%, and a balanced accuracy of 86.4%. However, its outer out-of-fold ROC-AUC was lower than that of the elastic-net model, at 0.800. The elastic-net/Extra Trees ensemble reached an outer out-of-fold ROC-AUC of 0.715. The Extra Trees classifier showed the lowest discrimination power, with an outer out-of-fold ROC-AUC of 0.649. The elastic-net logistic regression model was chosen as the primary model because it provided the best balance between discrimination, calibration, and stability during cross-validation. In contrast, tree-based methods exhibited larger differences between inner-CV and outer-fold performance, indicating greater instability in this small, highly imbalanced cohort.

### Explainability and radiomic feature contributions

The most informative features were derived from T1CE, T2, and FLAIR MRI, indicating that PTEN mutation classification was supported by a multisequence radiomic phenotype (**Table 3**). The top-ranked features included wavelet- and LoG-filtered first-order and texture descriptors, with prominent contributions from GLCM, NGTDM, and first-order feature classes.

**Table 3:** Top contributing radiomic feature families.

Feature	Modality	Filter	Feature class	Feature family
L3 T2 wavelet-LHH NGTDM Contrast	T2	Wavelet-LHH	NGTDM	Texture
L3 T2 original shape Flatness	T2	Original	Shape	Shape
L3 T1CE wavelet-LHL first-order Skewness	T1CE	Wavelet-LHL	First-order	Intensity
L3 FLAIR LoG sigma 3.0 mm first-order Maximum	FLAIR	LoG sigma 3.0 mm	First-order	Intensity
L3 FLAIR wavelet-LHL GLCM Correlation	FLAIR	Wavelet-LHL	GLCM	Texture
L3 T2 wavelet-LLH GLCM Inverse Variance	T2	Wavelet-LLH	GLCM	Texture
L3 FLAIR wavelet-HHL GLCM MCC	FLAIR	Wavelet-HHL	GLCM	Texture
L3 FLAIR LoG sigma 0.5 mm GLCM Cluster Shade	FLAIR	LoG sigma 0.5 mm	GLCM	Texture
L3 T1CE wavelet-LLH GLCM Inverse Variance	T1CE	Wavelet-LLH	GLCM	Texture
L3 T1CE wavelet-HHL GLCM Inverse Variance	T1CE	Wavelet-HHL	GLCM	Texture

**Figure 4:**
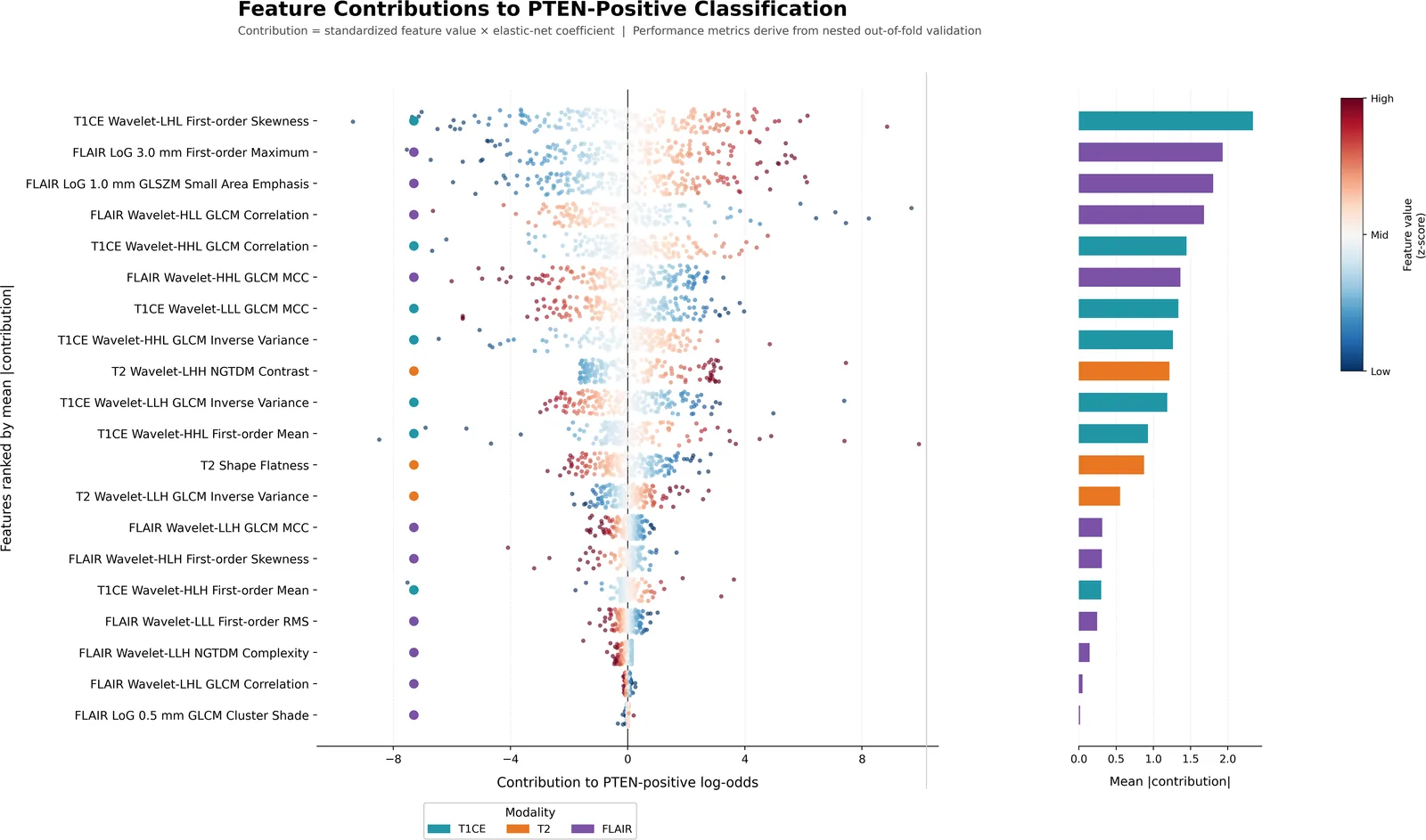
Linear SHAP-style feature contributions for the primary elastic-net PTEN radiomics model. Feature-level contributions were estimated as the standardized radiomic feature value multiplied by the corresponding elastic-net coefficient, providing an additive log-odds contribution toward PTEN-positive classification. Features are ranked by mean absolute contribution. Each point represents one patient’s contribution for a given feature, with point color indicating the patient’s standardized feature value (red = high, blue = low); the colored marker beside each feature name indicates the MRI modality. The most influential features were derived from T1CE, T2, and FLAIR sequences and included wavelet- and LoG-filtered first-order and texture features.

Linear SHAP-style feature contribution analysis showed that several high-ranking features contributed additively to PTEN-positive log-odds (**Figure 4**). The strongest contributors included T1CE wavelet-LHL first-order skewness, FLAIR LoG sigma 3.0 mm first-order maximum, FLAIR LoG sigma 1.0 mm GLSZM small-area emphasis, FLAIR wavelet-HLL GLCM correlation, T1CE wavelet-HHL GLCM correlation, FLAIR wavelet-HHL GLCM maximal correlation coefficient, T1CE wavelet-LLL GLCM maximal correlation coefficient, T1CE wavelet-HHL GLCM inverse variance, and T2 wavelet-LHH NGTDM contrast.

The predominance of GLCM and NGTDM texture features suggests that PTEN mutation status was associated with differences in spatial gray-level relationships, neighborhood intensity variation, and local texture organization within the enhancing tumor core. The contribution of wavelet- and LoG-filtered features further indicates that the model captured multiscale heterogeneity in the signal rather than relying solely on conventional first-order intensity or shape measurements.

T1CE-derived features suggest that heterogeneity in enhancement contributed to PTEN mutation classification. T2 and FLAIR-derived features indicate that non-enhancing signal heterogeneity and edema- or infiltration-associated tissue architecture also contributed to the radiomic phenotype. Together, these findings support a PTEN-associated imaging phenotype characterized by multisequence heterogeneity within the enhancing tumor core.

### Feature stability analysis

To evaluate the robustness of feature selection across cross-validation folds, we examined the overlap of selected radiomic features among the five outer folds. The model selected a mean of 50.0 ± 30.6 features per fold from the initial 3,669-feature pool. Across all folds, 130 unique features were selected, of which 38 were selected in at least 3 of 5 folds. The mean pairwise Jaccard similarity was 0.250 ± 0.129, while the Kuncheva stability index was 0.583 ± 0.227, indicating moderate feature-selection stability after accounting for the high-dimensional feature space. Several top contributors were repeatedly selected across folds, including T1CE wavelet-LHL first-order Skewness, selected in 5/5 folds, and FLAIR wavelet-LHL GLCM Correlation and FLAIR wavelet-HHL GLCM MCC, each selected in 4/5 folds.

### Radiomic phenotype summary

The primary elastic-net model identified a PTEN mutation-associated radiomic phenotype within the enhancing tumor core of a mixed post-treatment glioma cohort. This phenotype was characterized by multisequence texture and intensity heterogeneity across T1CE, T2, and FLAIR MRI. Although the model achieved strong ROC-AUC and high sensitivity in nested internal validation, the small PTEN-positive subgroup and modest PR-AUC indicate that these findings should be interpreted as exploratory and hypothesis-generating rather than clinically definitive.

### *Post-hoc* confounding analysis

To determine whether the radiomic score reflected differences related to age, treatment, or IDH status rather than PTEN biology, we performed several exploratory *post hoc* Firth-penalized logistic regression analyses. First, penalization was chosen due to the small number of PTEN-positive cases (n = 11). Age adjustment showed that the radiomic score remained independently associated with PTEN positivity after controlling for age (OR per SD: 1.59, 95% CI 1.11–2.29, p = 0.004). Age had a trend-level association (OR per SD: 1.96, 95% CI 0.78–4.96, p = 0.043 via penalized LRT). The classifier was trained solely on MRI features; age was not part of the model inputs. Adjusting for IDH status was not feasible due to complete separation, as all 11 *PTEN*-mutant cases were IDH-wildtype (adjusted OR 1.20, 95% CI 0.04–33.87 *p* = 1.000). This complete overlap reflects established tumor biology; previous radiogenomic studies have similarly evaluated cohorts dominated by these high-grade profiles, whether restricted strictly to glioblastoma, as in Li et al., or spanning all WHO grades, as in Chen et al. Consequently, this radiomic pattern should be interpreted as a signature of *PTEN* deficiency within the framework of IDH-wildtype disease, rather than a completely IDH-independent marker (11, 12). Regarding treatment and MRI timing, because imaging came from a postoperative surveillance database, we examined whether post-treatment exposure or scan timing affected the radiomic association. No significant differences were found between PTEN-positive and PTEN-negative groups in MRI timing or documented treatments—such as chemotherapy, radiation, immunotherapy, or other systemic therapies (all p > 0.29; **Table 1**, Supplement). The percentage of scans taken after chemotherapy or radiation was also balanced across groups (p = 0. 367 and p = 0.749). After adjusting for MRI timing, the radiomic score was still associated with PTEN positivity (OR per SD: 1.62, 95% CI: 1.13–2.31, p = 0.004). Similar results persisted after adjusting for treatment exposure and in a comprehensive model including age, IDH status, MRI timing, and treatment variables (OR per SD: 1.57, 95% CI: 1.08–2.27, p = 0.005). These results indicate that the radiomic association is independent of treatment differences or scan timing.

## Discussion

Our results demonstrate that conventional multiparametric MRI radiomics can detect an imaging phenotype linked to PTEN mutation status in gliomas. Our model achieved strong discrimination, with an outer out-of-fold ROC-AUC of 0.874. At the chosen threshold, the model showed high sensitivity for PTEN-positive cases, moderate specificity, and strong balanced accuracy. However, precision–recall performance was more modest, with a PR-AUC of 0.211 given a PTEN-positive prevalence of only 5.6%, which explains the low PPV in that setting. This divergence is important because ROC-AUC reflects the model’s ability to rank PTEN-positive cases above PTEN-negative cases across thresholds, whereas PR-AUC is more sensitive to minority-class prevalence and better reflects the reliability of positive predictions in imbalanced datasets. Therefore, the high ROC-AUC suggests that the radiomic score captures a PTEN-associated imaging signal, but the modest PR-AUC indicates that positive predictions remain uncertain and that the model is not yet suitable as a standalone clinical classifier. Therefore, our findings are exploratory and hypothesis-generating rather than definitive.

The explainability analysis indicates a biologically plausible PTEN-related imaging phenotype observed across T1CE, T2, and FLAIR MRI scans. The influence of T1CE skewness and texture features related to enhancement suggests that PTEN-mutant tumors may have altered enhancement patterns, possibly reflecting heterogeneous vascular permeability, hypoxia, necrosis, or poorly perfused tumor core components. This aligns with glioblastoma biology, in which hypoxia and necrosis are linked to HIF-1/VEGF-driven angiogenesis, and with PTEN pathology, in which dysregulation of PTEN/PI3K/AKT/mTOR pathways promotes tumor growth, angiogenesis, and tumor invasion (12, 20–23). FLAIR and T2 features, such as LoG-filtered maximum and GLSZM small-area emphasis, may indicate edema- or infiltration-related signal abnormalities and disruption of the enhancing tumor core’s tissue homogeneity (7). However, these associations are indirect and need validation with larger external datasets and molecular, transcriptomic, or histopathologic data.

Our results are comparable with the existing literature, although methods vary in glioma cohorts, imaging inputs, modeling approaches, and validation procedures. Li et al. (2019) developed an SVM classifier to predict PTEN mutations in glioblastoma using T1CE and T2-weighted MRI, achieving an AUC of 0.925 on the training set and 0.787 on the validation set. Their analysis also linked the imaging features to PTEN-related biological processes, such as angiogenesis, infiltration, edema, and tumor invasiveness (12). Chen et al. (2021) expanded on this using deep learning and radiomics models in a larger, mixed-grade glioma cohort, reporting AUCs of approximately 0.84 for the CNN, 0.83 for the radiomics model, and 0.91 for the combined model on the test set (11). Kazerooni et al. (2025) reported on using a deep learning (ResNet) and a machine learning (SVM) classifier to predict multiple molecular biomarkers in an IDH-Wildtype glioblastoma cohort. Specifically for PTEN co-occurring alterations, the deep learning model achieved an AUC of 0.71, while the SVM achieved 0.62 with conventional MRI sequences; with advanced sequences, performance improved to 0.86. Recently, Feng et al. (2026) introduced a spherical radiomics framework for 299 GBM patients and predicted multiple biomarkers. Additionally, more specifically, this approach achieved an AUC of 0.76 for predicting PTEN mutations using spatially structured features from concentric tumor shells, with GLCM texture descriptors being the most predictive. Although this method outperformed traditional Euclidean radiomics and included external validation, it relied on a specialized spherical decomposition pipeline (24). Compared to these studies, our model emphasizes an interpretable, leakage-aware traditional radiomics method that utilizes T1CE, T2, and FLAIR features from the tumor-enhancing core. While our internal AUC results are promising, caution is warranted in interpretation, given the notable PTEN-positive class imbalance and the lack of independent validation.

This study is clinically important and suggests that PTEN-focused radiogenomics can supplement, but not replace, tissue testing for gliomas, offering a noninvasive way to assess tumor heterogeneity from imaging. An MRI-based PTEN signature, once externally validated, could aid in risk assessment, prognosis, and trial eligibility, especially since PTEN/PI3K/AKT pathway abnormalities are associated with tumor progression and therapy resistance (25, 26). Recent research supports the broader role of imaging biomarkers in molecular profiling and trial stratification in IDH-wildtype glioblastoma (27). However, due to our retrospective study, lack of external validation, and PTEN class imbalance, this model remains hypothesis-generating and not ready for clinical use.

Our study has multiple strengths. First, we analyzed a diverse group of adult gliomas rather than focusing solely on glioblastoma, enabling us to assess whether PTEN-related radiomic patterns are present across a broader range of gliomas. Second, we employed a strict nested cross-validation process with leakage-aware preprocessing, redundancy removal, feature selection, scaling, and model tuning within the training data. This is especially crucial in high-dimensional radiomics, where data leakage and collinearity can lead to overestimated performance. Third, the model was based on routine clinical MRI sequences, including T1CE, T2, and FLAIR, supporting potential translatability without requiring advanced quantitative MRI sequences that are more sensitive to acquisition parameters, scanner differences, and post-processing variability. Lastly, the explainability analysis strengthens the case by clarifying how the model makes decisions and by highlighting a biologically plausible PTEN-related phenotype characterized by heterogeneity in enhancement, FLAIR, and T2 signal patterns, as well as local texture complexity.

On the other hand, several limitations should be acknowledged. First, this was a retrospective study, which may introduce selection bias related to patient inclusion, MRI availability, segmentation quality, and molecular testing patterns. Second, the model was evaluated using nested internal validation but lacked an independent external validation cohort. Therefore, its generalizability across institutions, scanners, acquisition protocols, and patient populations remains uncertain. Third, the cohort showed severe class imbalance, with only a small number of PTEN-positive cases. Although the model achieved strong ROC-AUC and high sensitivity, the precision–recall performance was modest, indicating limited positive-class reliability in this low-prevalence setting. In addition, with such a small minority class, individual PTEN-positive cases may have disproportionate influence on feature selection, coefficient estimation, threshold-dependent metrics, and cross-validation performance. Therefore, the findings should be interpreted as exploratory and hypothesis-generating, reflecting a possible PTEN-associated imaging phenotype rather than a clinically validated classifier. Fourth, although *post hoc* analyses adjusted for age, MRI timing, and treatment-related variables, complete adjustment for IDH status was not possible because all PTEN-positive tumors were IDH-wildtype, resulting in complete biological and statistical collinearity. Therefore, while the radiomic association was not explained by the available confounders, we cannot conclude that it is entirely independent of IDH-wildtype biology. Rather, the findings should be interpreted as a PTEN-associated imaging phenotype within the context of IDH-wildtype glioma. Larger, multi-institutional cohorts with higher PTEN-positive case counts and independent external validation are essential to verify the robustness and reproducibility of this radiomic signature. Future radiogenomic validation studies should be designed based on the number of PTEN-positive cases and the desired accuracy of the primary performance measure, rather than just total sample size. There is no universal minimum sample size for PTEN radiogenomic validation, as it depends on factors such as the expected PTEN-positive prevalence, the target confidence interval width, and whether ROC-AUC, PR-AUC, sensitivity, or positive predictive value is used as the primary endpoint. As a practical guideline, robust external validation would likely require 50–100 PTEN-positive cases, ideally more for precise performance estimation. Future research should aim to validate this method in larger, multi-institutional cohorts with more balanced PTEN-positive representation and incorporate molecular, transcriptomic, or histopathologic data to confirm the biological relevance of the radiomic phenotype.

## Conclusions

In summary, our study demonstrated that interpretable multiparametric MRI radiomics can identify a biologically plausible, PTEN-related imaging phenotype in adult gliomas. The primary elastic-net model demonstrated strong internal discrimination with an outer out-of-fold ROC-AUC of 0.874 and a balanced accuracy of 81.3%. A multisequence signature combining T1CE, T2, and FLAIR sequences is associated with altered enhancement patterns and disrupted local texture. The significance of this research lies more in its potential to offer a noninvasive, reproducible, and biologically meaningful approach to PTEN characterization than in the performance metrics alone. This method could eventually complement tissue-based testing, especially in post-treatment surveillance where repeat biopsies are difficult. However, limitations such as the small PTEN-positive sample size, significant class imbalance, modest PR-AUC, and lack of external validation indicate these findings are preliminary rather than definitive. Future studies should focus on external validation in larger, multi-institutional, PTEN-balanced cohorts, ideally including molecular or histopathologic data to better understand the biological relevance of the identified radiomic features.

## Data Availability

Publicly available datasets were analyzed in this study. This data can be found here: Repository: The Cancer Imaging Archive (TCIA) Dataset/collection name: University of Missouri Post-operative Glioma Dataset (MU-Glioma-Post) Persistent identifier/accession: DOI: 10.7937/7K9K-3C83 Direct link: https://www.cancerimagingarchive.net/collection/mu-glioma-post/.
