# Supplementary material for: Explainable MRI radiomics identifies a PTEN-associated imaging phenotype in adult gliomas
Source: Front Oncol. 2026 Aug 19;16:1900609. doi: 10.3389/fonc.2026.1900609 (PMC13533706; doi:10.3389/fonc.2026.1900609)
Supplement: Supplementary file 1 [file Supplementaryfile1.docx]

**Table S1. Exploratory assessment of MRI timing, treatment exposure, and adjusted association between radiomic score and PTEN positivity**

**A. MRI timing and treatment-related characteristics by PTEN status**

| **Variable** | **PTEN-negative (n = 184)** | **PTEN-positive (n = 11)** | **p value** |
| --- | --- | --- | --- |
| MRI timepoints, median | 2.0 | 2.0 | 1.000 |
| Mean MRI day from diagnosis, median, days | 137.5 | 121.0 | 0.299 |
| Initial chemotherapy documented | 118/184 (64.1%) | 8/11 (72.7%) | 0.750 |
| Radiation therapy documented | 128/184 (69.6%) | 8/11 (72.7%) | 1.000 |
| MRI after chemotherapy end | 106/184 (57.6%) | 8/11 (72.7%) | 0.367 |
| MRI after radiation end | 115/184 (62.5%) | 8/11 (72.7%) | 0.749 |
| Additional therapy documented | 101/184 (54.9%) | 5/11 (45.5%) | 0.553 |
| Immunotherapy documented | 48/184 (26.1%) | 3/11 (27.3%) | 1.000 |

**B. Adjustment models for the association between radiomic score and PTEN positivity**

| **Adjustment model** | **Radiomic score OR per SD** | **95% CI** | **p value** |
| --- | --- | --- | --- |
| Age + IDH | 1.59 | 1.11–2.29 | 0.004 |
| MRI timing | 1.62 | 1.13–2.31 | 0.004 |
| Post-treatment exposure | 1.60 | 1.12–2.30 | 0.004 |
| Age + IDH + MRI timing + post-treatment exposure, | 1.57 | 1.08–2.27 | 0.005 |

***Note:*** *Fisher’s exact test was used for categorical treatment-exposure comparisons. MRI timing variables were compared using the Mann–Whitney U test. Adjusted association models used Firth-penalized logistic regression, with PTEN status as the outcome. The radiomic score was standardized, and odds ratios are reported per 1-standard-deviation increase. Model sample sizes ranged from n = 180 to n = 194, depending on the availability of covariates. IDH adjustment should be interpreted cautiously because all PTEN-positive cases were IDH-wildtype, resulting in biological and statistical collinearity.*

**Table S2. Reproducible nested cross-validation workflow, hyperparameter search space, selected fold-level parameters, and fold-level performance**

**A. Pipeline steps**

| **Step** | **Method** |
| --- | --- |
| Input MRI sequences | T1CE, T2, and FLAIR |
| Segmentation label | Label 3 enhancing tumor core |
| Timepoint aggregation | Patient-level aggregation using the all_mean policy |
| Analytic cohort | 195 patients: 11 PTEN-positive and 184 PTEN-negative |
| Initial feature set | 3,669 label-3 T1CE/T2/FLAIR radiomic features |
| Missingness filtering | Features exceeding the missingness threshold were removed within training folds |
| Variance filtering | Zero- and near-zero-variance features were removed within training folds |
| Redundancy pruning | Spearman correlation-based pruning performed within training folds |
| Univariate filtering | Mann–Whitney U ranking performed within training folds |
| Model-based reduction | Elastic-net/permutation-feature-importance ranking performed within training folds |
| Final feature set | Top-k selected features within each outer training fold |
| Class-imbalance handling | Majority-class downsampling performed within training folds only |
| Scaling | Feature scaling fit within training folds and applied to validation/test folds |
| Primary model | Elastic-net logistic regression |
| Validation strategy | Patient-level nested cross-validation with 5 outer folds and 3 inner folds |
| Hyperparameter tuning | Optuna optimization within inner cross-validation |
| Optimization metric | ROC-AUC |
| Confidence intervals | Bootstrap resampling of pooled outer out-of-fold predictions |
| Leakage prevention | No preprocessing, feature selection, resampling, calibration, threshold selection, or hyperparameter tuning used outer-fold test data |

**B. Hyperparameter search space**

| **Hyperparameter** | **Search range** |
| --- | --- |
| Spearman correlation threshold | 0.60, 0.70, 0.80, 0.90 |
| Mann–Whitney retained features | 50, 100, 200, 400, 800 |
| Final top-k features | 10, 25, 50, 100 |
| Negative-to-positive downsampling ratio | 1:1, 2:1, 3:1, 5:1 |
| Elastic-net inverse regularization strength, C | 0.001 to 100, log scale |
| Elastic-net l1_ratio | 0.0 to 1.0 |
| Class weight | None |
| Tuning algorithm | Optuna |
| Optimization metric | ROC-AUC |

**C. Selected hyperparameters by outer fold**

| **Outer fold** | **Spearman threshold** | **Features retained after Spearman pruning** | **Mann–Whitney top-k** | **Features retained after Mann–Whitney filtering** | **Final top-k** | **Negative:positive downsampling ratio** | **Elastic-net C** | **Elastic-net l1_ratio** |
| --- | --- | --- | --- | --- | --- | --- | --- | --- |
| 1 | 0.60 | 85 | 800 | 85 | 25 | 3:1 | 10.3068 | 0.0640 |
| 2 | 0.80 | 331 | 200 | 200 | 25 | 2:1 | 26.7969 | 0.1698 |
| 3 | 0.80 | 346 | 100 | 100 | 50 | 5:1 | 27.8305 | 0.4755 |
| 4 | 0.80 | 345 | 100 | 100 | 50 | 3:1 | 25.5828 | 0.5232 |
| 5 | 0.80 | 336 | 400 | 336 | 100 | 3:1 | 3.1328 | 0.4596 |

**D. Fold-level performance**

| **Outer fold** | **PTEN-positive cases in test fold** | **PTEN-negative cases in test fold** | **Fold ROC-AUC** | **Fold PR-AUC** | **Selected threshold** |
| --- | --- | --- | --- | --- | --- |
| 1 | 2 | 37 | 0.973 | 0.750 | 0.0517 |
| 2 | 2 | 37 | 0.811 | 0.238 | 0.0507 |
| 3 | 2 | 37 | 0.865 | 0.226 | 0.0547 |
| 4 | 2 | 37 | 0.851 | 0.208 | 0.0648 |
| 5 | 3 | 36 | 0.907 | 0.567 | 0.0503 |

**Algorithm S1. Nested cross-validation radiomics pipeline for PTEN mutation classification**

**Input:** Patient-level T1CE, T2, and FLAIR radiomic features from label 3, corresponding to the enhancing tumor core; binary PTEN mutation status.
**Output:** Outer-fold out-of-fold predictions, calibrated probabilities, selected features, fold-level hyperparameters, operating thresholds, and pooled model-performance metrics.

**Algorithm**

1. **Cohort definition**
   1. Define the analytic cohort as patients with available label-3 radiomic features and known binary PTEN mutation status.
   2. Aggregate repeated timepoint-level radiomic features to patient-level features using the predefined all_meantimepoint policy.
2. **Outer cross-validation**
   1. Split the cohort into five stratified outer cross-validation folds at the patient level.
   2. For each outer fold:
      1. Assign the outer training set and the held-out outer test set.
      2. Use the outer training set only for preprocessing, feature selection, hyperparameter tuning, calibration, and threshold selection.
3. **Inner-loop hyperparameter tuning**
   1. Within each outer training set, create three stratified inner cross-validation folds.
   2. For each Optuna hyperparameter trial:
      1. Select candidate hyperparameters, including:
         - Spearman correlation threshold
         - Mann–Whitney retained-feature count
         - Final top-k feature count
         - Majority-class downsampling ratio
         - Elastic-net C
         - Elastic-net l1_ratio
      2. For each inner fold:
         1. Fit all preprocessing and feature-selection steps using only the inner-training data.
         2. Remove features exceeding the missingness threshold.
         3. Impute retained features using median imputation fit on the inner-training data.
         4. Remove zero- and near-zero-variance features.
         5. Apply Spearman correlation-based redundancy pruning.
         6. Rank retained features using Mann–Whitney U statistics.
         7. Perform model-based feature ranking using L1 logistic-regression coefficients and permutation feature importance within the inner-training data.
         8. Select the final top-k features.
         9. Fit the scaler on the selected inner-training features and apply it to the inner-validation features.
         10. Downsample the majority class within the scaled inner-training data only.
         11. Train the elastic-net logistic regression model.
         12. Apply prior-probability correction after downsampling.
         13. Evaluate inner-validation ROC-AUC.
      3. Compute the mean inner-validation ROC-AUC for the trial.
   3. Select the hyperparameter set with the highest mean inner-validation ROC-AUC.
4. **Outer-training calibration and threshold selection**
   1. Using only the outer training set and the selected hyperparameters, generate inner out-of-fold raw predictions.
   2. Fit a logistic calibration model using these outer-training inner out-of-fold predictions.
   3. Select the operating threshold using Youden’s index on calibrated outer-training inner out-of-fold predictions.
   4. The held-out outer test fold is not used for calibration or threshold selection.
5. **Outer-fold model fitting and testing**
   1. Refit the complete preprocessing, feature-selection, scaling, downsampling, and elastic-net model pipeline on the full outer training set using the selected hyperparameters.
   2. Apply the fitted pipeline to the held-out outer test set.
   3. Apply the fold-specific logistic calibrator to the outer-test predicted probabilities.
   4. Apply the fold-specific Youden threshold to obtain binary predictions.
   5. Store the following for each outer fold:
      1. Raw predicted probabilities
      2. Calibrated predicted probabilities
      3. Binary predictions
      4. Selected features
      5. Selected hyperparameters
      6. Operating threshold
      7. Fold-level performance metrics
6. **Pooled performance estimation**
   1. Pool calibrated outer-fold out-of-fold predictions across all patients.
   2. Calculate final model-performance metrics, including:
      1. ROC-AUC
      2. PR-AUC
      3. Brier score
      4. Sensitivity
      5. Specificity
      6. Positive predictive value
      7. Negative predictive value
      8. Balanced accuracy
      9. Confusion-matrix counts
   3. Estimate confidence intervals using bootstrap resampling of pooled outer-fold out-of-fold predictions.
7. **Post hoc model interpretation**
   1. Assess feature-selection stability across outer folds.
   2. Perform explainability analysis using the selected radiomic features and pooled out-of-fold predictions.

**Leakage-control rule**

Missingness filtering, median imputation, variance filtering, Spearman redundancy pruning, Mann–Whitney ranking, L1/permutation-feature-importance ranking, final feature selection, scaling, majority-class downsampling, hyperparameter tuning, calibration, and threshold selection were performed using training data only within each fold. The held-out outer test folds were used only for final out-of-fold prediction and performance estimation.
